# SVScore: an impact prediction tool for structural variation

**DOI:** 10.1093/bioinformatics/btw789

**Published:** 2017-01-03

**Authors:** Liron Ganel, Haley J Abel, FinMetSeq Consortium, Ira M Hall

**Affiliations:** 1 McDonnell Genome Institute, Washington University School of Medicine, St. Louis, MO, USA; 2 Department of Medicine, Washington University School of Medicine, St. Louis, MO, USA

## Abstract

**Summary:**

Here we present SVScore, a tool for *in silico* structural variation (SV) impact prediction. SVScore aggregates per-base single nucleotide polymorphism (SNP) pathogenicity scores across relevant genomic intervals for each SV in a manner that considers variant type, gene features and positional uncertainty. We show that the allele frequency spectrum of high-scoring SVs is strongly skewed toward lower frequencies, suggesting that they are under purifying selection, and that SVScore identifies deleterious variants more effectively than alternative methods. Notably, our results also suggest that duplications are under surprisingly strong selection relative to deletions, and that there are a similar number of strongly pathogenic SVs and SNPs in the human population.

**Availability and Implementation:**

SVScore is implemented in Perl and available freely at {{http://www.github.com/lganel/SVScore}} for use under the MIT license.

**Supplementary information:**

Supplementary data are available at *Bioinformatics* online.

## 1 Introduction

Structural variation (SV) is an important source of human genome variation that includes deletions, duplications, inversions, mobile element insertions, translocations, and complex rearrangements. Over the past several years, much progress has been made in the area of SV detection, and we are now able to routinely detect 5000–10 000 SVs in a typical deeply sequenced human genome (Sudmant *et al.*, 2015a). However, predicting the functional impact of SVs discovered in whole genome sequencing (WGS) studies remains extremely challenging. Accurate SV impact prediction is especially important for WGS-based rare variant association studies and WGS-based studies of rare disease.

There have been many efforts to predict the effects of single nucleotide polymorphisms (SNPs), including SIFT (Ng and Henikoff, 2001), PolyPhen (Adzhubei *et al.*, 2010) and VEP (McLaren *et al.*, 2010). More recent tools such as fitCons (Gulko *et al.*, 2015), CADD (Kircher *et al.*, 2014) and Eigen (Ionita-Laza *et al.*, 2016) precompute pathogenicity scores for hypothetical variants at each base in the genome.

Constructing similar methods for SV is difficult due to the diversity of variant size and type. Variant type is important because, for example, a deletion spanning an entire gene is likely to have vastly different functional consequences than an inversion with the same coordinates. Furthermore, current sequencing technologies make precise SV breakpoint detection difficult, resulting in uncertainty about their exact location. SV impact prediction methods must take these all of these factors into consideration in order to robustly prioritize pathogenic variants.

There have been cursory attempts at SV impact prediction in the past. ANNOVAR (Yang and Wang, 2015) annotates previously reported CNVs and names overlapping genes, but does not make pathogenicity predictions, nor does it handle balanced rearrangements. VEP performs superficial consequence prediction for SVs, but only for a limited range of variant types (insertions, deletions and duplications). No existing method provides a quantitative SV pathogenicity score.

## 2 Methods

**Fig. 1.**
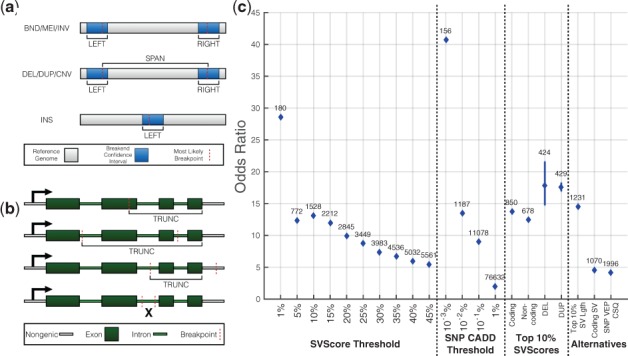
SVScore strategy and performance. (**a**) LEFT, RIGHT and SPAN intervals chosen by SVScore based on SV type. LEFT and RIGHT scores comprise the entire confidence interval (CI) around the left and right breakpoint, respectively, and are calculated for every variant type. Here, MEI refers specifically to mobile element insertions detected in the reference genome, whereas INS refers to all insertion types detected in the experimentally sequenced genome. For deletions (DEL), tandem duplications (DUP) and other copy number variants (CNV), a SPAN score is calculated using the interval between the most likely breakpoints. (**b**) Truncation scores (LTRUNC when truncated by the left breakend and RTRUNC otherwise) are calculated for deletions, inversions (INV), mobile element insertions (MEI) and INS variants that are predicted to truncate a transcript. (**c**) Above each point is the number of rare, pathogenic variants using the pathogenicity definition on the *x*-axis. The **SVScore Threshold** section shows the odds ratios for pathogenic SVs being rare under varying definitions of pathogenicity based on impact score percentile, where variants in the bottom 50% were considered benign. The **SNP CADD Threshold** section shows odds ratios calculated for SNPs using CADD at the percentile thresholds shown. For these odds ratios, SNPs with CADD scores in the bottom 50% were used as benign variants. Pathogenic variants used for calculations in the **Top 10% SVScores** section were all subsets of those SVs with impact scores in the top 10%. In this section, the variants in the bottom 50% of all impact scores were again called benign. For the ‘Coding’ and ‘Noncoding’ experiments, the pathogenic variants were those SVs in the top 10% of impact scores that did and did not overlap a refGene exon, respectively. In the ‘DEL’ and ‘DUP’ experiments, the pathogenic variants were DELs and DUPs, respectively, in the top 10% of scores. The size distributions of these variants were matched as described in Supplementary Methods, and the 95% confidence intervals are shown. The **Alternatives** section shows three odds ratios from SVScore alternatives. In the ‘Top 10% SV Lgth’ experiment, pathogenic variants were those with lengths at or above the 90th percentile, and benign variants were those below the 50th percentile. For ‘Coding SV’, pathogenic variants were those with at least one overlap between refGene exon and either a breakpoint CI or a SPAN interval, and benign variants were all others. The ‘SNP VEP CSQ’ experiment used VEP’s IMPACT predictions for SNPs—variants with at least one HIGH prediction on a canonical transcript were called pathogenic, while those with only LOW or MODIFIER predictions on canonical transcripts were categorized as benign. BND—‘unclassified’ structural variant

We present SVScore, a novel computational tool for *in silico* SV impact prediction. SVScore depends on an existing set of per-base pathogenicity scores; here we use the precomputed SNP scores from CADD v1.3, although any other scoring scheme could potentially be used. For each SV in an SV callset described in Variant Call Format (VCF), SVScore uses vcfanno (Pedersen *et al.*, 2016) to annotate the variant with overlapping gene and exon annotations. Next, it uses tabix (Li, 2011) to aggregate the chosen per-base scores across a set of genomic intervals (see Fig. 1) and applies an operation (e.g. max or sum, see below) to each interval to summarize the per-base scores into interval scores. One score is computed for each interval-operation pair.

The operations currently supported are: maximum, sum, mean and mean of the top N scores. The maximum of all of a variant’s interval scores is reported as the score for the given operation and added to the INFO column of the VCF line(s). SVScore supports weighting scores using probability distributions calculated by tools such as LUMPY (Layer *et al.*, 2014). These give the probability of the true breakpoint being located at each possible position in a CI, which is important to consider because bases at a tail of the breakpoint probability distribution are less likely to represent the true breakpoint than bases at the center of the distribution (see Supplementary Methods).

## 3 Results and discussion

To evaluate SVScore’s computational performance, we computed scores for a set of high confidence SVs called from 950 Finnish WGS datasets (see Supplementary Methods). Scores were calculated using SVScore v0.5.1 with 5 operations—maximum, sum, weighted mean and weighted mean of the top 10 and 100 bases in each interval. On a machine with two Intel Xeon E5-2670 processors (each with 16 threads) and 128 GB RAM, the total CPU time was 341 min. With 21 426 SVs passing all of our filters, the average time per variant was 1.01 s. The average memory used was 1.7 GB, and the maximum memory was 3.5 GB (see Supplementary Fig. S1 and Supplementary Table S1).

To evaluate SVScore’s effectiveness in predicting deleterious variants, we used population allele frequency as a proxy for pathogenicity. Due to the effects of purifying selection, strongly pathogenic variants are likely to be observed at very low frequency in the human population. Thus, if SVScore is an accurate predictor of pathogenicity, the variants it predicts to be deleterious should be significantly more rare than those it predicts to be benign. For this experiment, impact scores were calculated using the weighted mean of the top 10 bases in each interval and exon/intron annotations from refGene. Supplementary Figure S2 shows the allele frequency spectra of ‘pathogenic’ (impact scores at or above the 90th percentile), ‘benign’ (below the 50th percentile) and ‘intermediate’ variants (all others). The predicted pathogenic variants were heavily skewed toward the rare (AF < 0.01) end of the spectrum, while predicted benign variants were heavily skewed toward the common (AF ≥ 0.05) end, and variants with intermediate scores were between the other two categories. The difference between pathogenic and benign SVs was highly significant (rare versus common OR = 13.06, *P* = $5.43\times 10^{-323}$, Fisher’s Exact Test). This suggests that high-scoring SVs are under strong purifying selection relative to low-scoring SVs, which strongly supports the utility of our impact scoring strategy. We calculated this odds ratio for several other definitions of ‘pathogenic’ and ‘benign’, (see Supplementary Table S2, Fig. 1c). Notably, coding SVs in the top 10% of impact scores had a greater odds ratio than noncoding variants in the same subset (see Fig. 1c), but the magnitude of this difference is surprisingly mild and suggests that many non-coding SVs are under similarly strong selection as coding SVs. Also, we found that even when controlling for size, the odds ratio for tandem duplications with impact scores in the top 10% was nearly equal to that for duplications. This result may suggest that duplications are under stronger selection than previously thought (Conrad *et al.*, 2006; Cooper *et al.*, 2011; Sudmant *et al.*, 2015a, b). Alternatively, this result may reflect ascertainment bias against pathogenic deletions that cause embryonic lethality or severe developmental defects, and thus were not present in our adult cohort. Further work will be required to disentangle these factors.

We next compared SVScore to two commonly used alternative methods (see **Alternatives** section of Fig. 1c). First, we defined pathogenicity based on whether or not a structural variant (in any of its LEFT, RIGHT, or SPAN intervals) overlapped an annotated exon. This analysis is similar to the SV consequence prediction offered by VEP. This method identified fewer rare, pathogenic SVs (1070 versus 1528) and resulted in a lower odds ratio than using the top 10% of impact scores.

As a second approach, we used SV length percentile alone as a predictor of pathogenicity, categorizing large SVs (top 10%) as pathogenic and small SVs (bottom 50%) as benign. This yielded an odds ratio of 14.46, which is slightly greater than the odds ratio of 13.06 when using the top 10% of impact scores as ‘pathogenic’; however, substantially fewer rare, pathogenic variants were identified using the SV length method (1231 versus 1528). Supplementary Figure S4 shows size distributions for structural variants in our callset. As impact scores increase, size distributions shift toward larger variants. However, there is considerable overlap between the distributions, suggesting that SVScore captures more information than length alone. Also, the latter method cannot be easily applied to translocations or other complex variants for which ‘length’ is undefined.

We next sought to calibrate our SV impact scoring method with existing SNP scoring methods. We first used IMPACT annotations from VEP to define pathogenicity of SNPs in our callset. This approach was far less effective than SVScore in discriminating between pathogenic and benign variants. Comparison of SVScore with CADD-based SNP impact scores revealed that the top 10% of highest scoring SVs (*N* = 1528) have a similarly strong allele frequency skew as the top 0.01% of SNPs (*N* = 1187). Interestingly, this result suggests that there may be a similar number of strongly pathogenic SVs and SNPs in the human population, despite the fact that SNPs are nearly 3 orders of magnitude more abundant overall.

Finally, we sought to further assess SVScore’s ability to discern common polymorphisms from pathogenic variation. To this end, we ran SVScore on a manually curated list of approximately 300 CNVs believed to be pathogenic from ClinGen (the iscaCuratedPathogenic track in the UCSC Genome Browser) as well as the full set of high-confidence SVs detected in phase 3 of the 1000 Genomes Project (Sudmant *et al.*, 2015a). The results, shown in Supplementary Figure S5, demonstrate that the distribution of ClinGen impact scores is shifted heavily to the right relative to that of 1000 Genomes variants.

A limitation of our method is that it depends on per-base SNP pathogenicity scores, and thus does not account for all mechanisms whereby SVs may be phenotypically impactful. SVScore does not optimally address gain-of-function mutations such gene fusions or novel adjacency with cis-regulatory elements.

SVScore will be useful for future WGS-based studies by enabling facile prioritization of SVs based on their likelihood of being deleterious. Its support for various operations and arbitrary per-base scoring schemes make it a powerful and flexible asset to investigators interested in the genetic variants underlying both Mendelian and complex phenotypes.

## Supplementary Material

Supplementary Data
