# Supplementary material for: SVScore: an impact prediction tool for structural variation
Source: Bioinformatics. 2017 Jan 3;33(7):1083–5. doi: 10.1093/bioinformatics/btw789 (PMC5408916; doi:10.1093/bioinformatics/btw789)
Supplement: Supplementary Data [file btw789_supp.zip › SVScore_Bioinformatics_SupplementaryMethods.docx]

**FinMetSeq Consortium**

Abel, Haley J^1,2^;Boehnke, Michael^3^;Chen, Lei^2^;Chiang, Charleston WK^4^;Chiang, Colby C^2^;Dutcher, Susan K^1,2^;Freimer, Nelson B^4^;Fulton, Robert S^2^;Hall, Ira M^2^;Havulinna, Aki S^5,6^;Jackson, Anne U^3^;Kanchi, Krishna L^2^;Kang, Chul Joo^2^;Koboldt, Daniel C^2,7,8^;Kurki, Mitja I^5,9,10^;Laakso, Markku^11^;Laivuori, Hannele^5,12,13^;Larson, David E^1,2^;Locke, Adam E^2,3^;Meltz Steinberg, Karyn^2^;Nelson, Joanne^2^;Nicholas, Thomas J^2^;Palotie, Aarno^5,14,15^;Pietilä, Arto^6^;Pirinen, Matti^5,16,17^;Ramensky, Vasily^4^;Ray, Debashree^3^;Ripatti, Samuli^5,16,18^;Sabatti, Chiara^19,20^;Salomaa, Veikko^6^;Scott, Laura J^3^;Service, Susan^4^;Stell, Laurel^19^;Stitziel, Nathan O^2,21^;Stringham, Heather M^3^;Welch, Ryan^3^;Wilson, Richard K^2,7,22^;Yajnik, Pranav^3^

^1^Department of Genetics, Washington University School of Medicine, St. Louis, MO;^2^McDonnell Genome Institute, Washington University School of Medicine, St. Louis, MO;^3^Department of Biostatistics and Center for Statistical Genetics, University of Michigan School of Public Health, Ann Arbor, MI;^4^Center for Neurobehavioral Genetics, Semel Institute for Neuroscience and Human Behavior, University of California Los Angeles, Los Angeles, CA;^5^Institute for Molecular Medicine Finland (FIMM), University of Helsinki, Helsinki, Finland;^6^National Institute for Health and Welfare, Helsinki, Finland;^7^Department of Pediatrics, The Ohio State University College of Medicine, Columbus, OH;^8^The Institute for Genomic Medicine, Nationwide Children's Hospital, Columbus, OH;^9^Program in Medical and Population Genetics, Broad Institute of MIT and Harvard, Cambridge, MA;^10^Psychiatric & Neurodevelopmental Genetics Unit, Departments of Psychiatry and Neurology, Massachusetts General Hospital, Boston, MA;^11^Institute of Clinical Medicine, Internal Medicine, University of Eastern Finland, Kuopio, Finland;^12^Medical and Clinical Genetics, University of Helsinki and Helsinki University Hospital, Helsinki, Finland;^13^Obstetrics and Gynecology, University of Helsinki and Helsinki University Hospital, Helsinki, Finland;^14^Analytical and Translational Genetics Unit (ATGU), Psychiatric & Neurodevelopmental Genetics Unit, Departments of Psychiatry and Neurology, Massachusetts General Hospital, Boston, MA;^15^Broad Institute of MIT and Harvard, Cambridge, MA;^16^Department of Public Health, University of Helsinki, Helsinki, Finland;^17^Helsinki Institute for Information Technology HIIT and Department of Mathematics and Statistics, University of Helsinki, Helsinki, Finland;^18^Wellcome Trust Sanger Institute, Hinxton, Cambridge, UK;^19^Department of Biomedical Data Science, Stanford University, Stanford, CA;^20^Department of Statistics, Stanford University, Stanford, CA;^21^Cardiovascular Division, Department of Medicine, Washington University School of Medicine, St. Louis, MO;^22^Institute for Genomic Medicine, Nationwide Children's Hospital, Columbus, OH

**Score Aggregation from Intervals**

Figure 1a shows how intervals are chosen for each supported variant type. While SPAN scores are calculated by aggregating per-base scores directly, the LEFT and RIGHT scores aggregate *possible breakpoint scores*, which are defined as the average of the scores of the 2 bases immediately flanking each possible breakpoint. If a breakpoint location is known precisely, the possible breakpoint score for this location is directly reported as the interval score regardless of the operation.

**Truncation Scores**

Truncation scores reflect the ability of certain SV types to truncate transcripts regardless of variant length (e.g. by disrupting an exon). Figure 1b shows how truncation scores are calculated. These scores are designated LTRUNC or RTRUNC depending on which breakpoint is involved. The intervals across which these scores are calculated extend from the truncating breakpoint to the furthest downstream base of the affected transcript. SVScore uses vcfanno v0.0.11 (Pedersen *et al*., 2016) to find exons or introns that overlap SVs. As with SPAN scores, truncation scores are calculated from per-base scores rather than possible breakpoint scores.

**Probability Distribution Weighting**

Weighting the possible breakpoint scores using these distributions is important for two reasons. First, the expected score scales with size for the maximum and sum operations, causing a bias toward variants with large CIs. However, these variants are simply detected imprecisely, which is unrelated to their true pathogenicity. The second reason is that bases at a tail of the breakpoint probability distribution should not be given the same weight as those in the center of the distribution, as the former bases are less likely to be truly affected. When probability distributions are available, SVScore can incorporate them into the calculations of mean scores. If they are not present, SVScore simply assumes a uniform distribution over the CI. For weighted means of the top N bases in each interval, possible breakpoint scores are first weighted by the probability distribution, then the top N are chosen and the probability distribution over the chosen bases is rescaled to sum to 1.

Probability distribution weighting is only available when using the overall mean or the mean of the top N bases. Otherwise, weighting LEFT and RIGHT scores unfairly biases the scores toward dosage altering variants. These variants have a SPAN score that is unweighted by any probability distribution (as there is no probability distribution across a SPAN) and thus likely to be greater than LEFT and RIGHT scores of balanced rearrangements. However, the weighted mean of a breakpoint CI is similar in scale to the unweighted mean of a SPAN, making these comparisons fair.

**Size Distribution Matching**

To compare the odds ratios for the deletions and tandem duplications within the top 10% of SVs by impact score (Figure 1c, “DEL” and “DUP”), we defined 49 logarithmically sized length bins between 1 and 10^6^, and added a 50^th^ bin for lengths greater than 10^6^. The edges of these bins were defined by $b^{n}$, where b =$\sqrt[49]{{10}^{6}}\approx1.326$ and n = 0,1,2,…,49. We then placed the deletions and duplications in the appropriate bins according to their lengths. Within each size bin, we compared the number of deletions ($x$) and duplications ($y$) present, and sampled $min(x,y)$ variants without replacement from both the deletions and duplications in that bin. We then aggregated all sampled deletions and all sampled duplications and calculated the odds ratio for each as in equation (1), using the bottom 50% of SVs as benign variants. This process was repeated 100,000 times, and the mean of the calculated odds ratios was reported for each variant type. The 95% confidence intervals shown in Figure 1c were drawn from the 2.5^th^ percentile of the odds ratio distribution to the 97.5^th^ percentile.

To compare the distributions of impact scores for known pathogenic variants from ClinGen and 1000 Genomes Phase 3 SVs or FinMetSeq SVs, a similar procedure was used to adjust the size distributions. First, any variants with a score of 100 (meaning that the variant length was greater than 1 Mbp) were excluded, leaving 261 variants. These SVs were then binned by size as before, and $min(x,y)$ SVs were again sampled without replacement from both the comparison data set (1000 Genomes or FinMetSeq) and the ClinGen variants. In this case, however, this sampling was repeated 1000 times, and the sampled variants (totaling 258,000 from each) were aggregated. Supplementary Figure 5 shows histograms of the impact scores of these sampled variants.

**SNP Callset Generation**

### ***Read Alignment***

Data were aligned in aggregate using SpeedSeq's (Chiang *et al*.,2015; gms branch - <https://github.com/hall-lab/speedseq/tree/gms> commit 1aa63c99b02d76db58db1182efe450b27f98e819) realign command. Briefly, each individual lane or sub-lane was stored as an unaligned BAM file containing read group information. For each possible library, SpeedSeq was used to convert each BAM to interleaved FASTQ and align it by streaming through mbuffer (v20140302), bwa mem (Li and Durbin, 2009; v0.7.10; -t 8 -C -p), samblaster (Faust and Hall, 2014; v0.1.22; --excludeDups --addMateTags --maxSplitCount 2 --minNonOverlap 20) and sambamba (Tarasov *et al*., 2015; v0.5.4) for BAM conversion (sambamba view) and sorting (sambamba sort). Both discordant and split-read containing BAM files were stored for later analysis by LUMPY (Layer *et al*., 2014). Python (v2.7) scripts that are part of SpeedSeq were utilized for BAM to FASTQ conversion, header addition and read group addition.

### ***Duplicate Read Marking and Merging***

Duplicates were marked by samblaster during alignment (but not included in the splitter and discordant BAM files). In the case of multiple libraries, a single BAM file was created using sambamba merge to merge the aligned BAMs from each individual library into a single file.

### *Individual-level Variant Calling*

Variant calls were generated using GATK (DePristo *et al*., 2011) HaplotypeCaller (v3.4; -ERC GVCF -GQB 5 -GQB 20 -GQB 60 -variant_index_type LINEAR -variant_index_parameter 128000) parallelized into 13 groups of chromosomes empirically chosen to have approximately equal run times. Within each group of chromosomes, each chromosome was run in serial.

### *Cohort-level Variant Calling*

GVCFs containing SNVs and Indels from GATK HaplotypeCaller were combined (CombineGVCFs), genotyped (GenotypeGVCFs; -stand_call_conf 30 -stand_emit_conf 0), variant score recalibrated (VariantRecalibrator) and filtered (ApplyRecalibration) using GATK (v3.4). SNP variant recalibration was performed using the following options to VariantRecalibrator and all resources were drawn from the GATK resource bundle (v2.5):

- -mode SNP
- -resource:hapmap,known=false,training=true,truth=true,prior=15.0
- -resource:omni,known=false,training=true,truth=true,prior=12.0
- -resource:1000G,known=false,training=true,truth=false,prior=10.0
- -resource:dbsnp,known=true,training=false,truth=false,prior=2.0
- -an QD -an DP -an FS -an MQRankSum -an ReadPosRankSum
- -tranche 100.0 -tranche 99.9 -tranche 99.0 -tranche 90.0

Indel variant recalibration was performed using the following options to VariantRecalibrator (with the same resource bundle as with SNPs):

- -mode INDEL
- -resource:mills,known=true,training=true,truth=true,prior=12.0
- -an DP -an FS -an MQRankSum -an ReadPosRankSum
- --maxGaussians 4
- -tranche 100.0 -tranche 99.9 -tranche 99.0 -tranche 90.0

When applying the variant recalibration the following options were used:

- For SNPs: --ts_filter_level 99.9
- For Indels: --ts_filter_level 99.0

Subsequently, the resulting VCFs were processed to remove alternate alleles where the allele count was 0 in the cohort (GATK SelectVariants –removeUnusedAlternates). The remaining calls were then processed using vt (Tan *et al*., 2015; v0.5) to decompose multi-allelic variants (vt decompose -s), normalize indel representations (vt normalize), and remove duplicate calls (vt uniq). After processing with vt, sites where >2% of samples were missing genotypes were removed using a Python script. Sites previously discovered in the 1000 Genomes Project phase 3 call set were annotated using bcftools (Li, 2011; v1.2) annotate (-a ALL.wgs.phase3_shapeit2_mvncall_integrated_v5.20130502.sites.decompose.normalize.reheader.w_ids.vcf.gz –c ID) and gene annotation added using the Ensembl Variant Effect Predictor (v76; --force_overwrite --offline --fork 12 --cache --dir_cache $VEP_CACHE --dir_plugins $LOFTEE_PLUGIN --plugin LoF,human_ancestor_fa:$LOFTEE_PLUGIN/human_ancestor.fa.gz,conservation_file:$LOFTEE_PLUGINphylocsf.sql --sift b --polyphen b --species homo_sapiens --symbol --numbers --biotype --total_length -o STDOUT --format vcf --vcf --fields Consequence,Codons,Amino_acids,Gene,SYMBOL,Feature,EXON,PolyPhen,SIFT,Protein_position,BIOTYPE,LoF,LoF_filter,LoF_flags,LoF_info --no_stats). Analysis was limited to SNPs with VQSLOD > 0.8150.

**SV Callset Generation**

Cohort-level structural variant calls were produced using the SpeedSeq SV pipeline (Chiang *et al*., 2015), followed by the svtools package (v0.2.0; <https://github.com/hall-lab/svtools>). Briefly, speedseq sv, which comprises LUMPY for SV calling based on discordant pairs and split-reads; svtyper (Chiang *et al*., 2015) for SV genotyping; and cnvnator (Abyzov *et al*., 2011) for read-depth based CNV detection; was run on each sample individually. The individual-level calls were sorted and merged using svtools lmerge, and then each sample was re-genotyped and copy number annotated at all variant positions using svtools genotype and copynumber, and pasted into a single cohort-level VCF. svtools classify was used to reclassify putative deletions and duplications based on the read-depth information as well as to annotate calls overlapping known LINE and SINE elements. Finally, inversion calls and unclassified novel adjacencies (i.e. BNDs) were subjected to an additional filter: inversion calls in which fewer than 10% of reads supported either breakpoint or in which either discordant pairs or split-reads represented less than 10% of the evidence supporting the variant were excluded. Likewise for BNDs: calls where either paired-end or split-read evidence comprised less than 25% of the evidence supporting a variant were flagged as low quality.

**References**

Abyzov A. *et al*. (2011) CNVnator: an approach to discover, genotype, and characterize typical and atypical CNVs from family and population genome sequencing. *Genome Res*, **6**, 974-984.

Chiang C. *et al*. (2015) SpeedSeq: ultra-fast personal genome analysis and interpretation. *Nat Methods*, **12**, 966-968.

DePristo *et al*. (2011) A framework for variation discovery and genotyping using next-generation DNA sequencing data. *Nat Genet*, **43**, 491-498.

Faust,G.G. and Hall,I.M. (2014) SAMBLASTER: fast duplicate marking and structural variant read extraction. *Bioinformatics*, **30**, 2503-2505.

Layer,R.M. *et al*. (2014) LUMPY: a probabilistic framework for structural variant discovery. *Genome Biol*, **15**, R84.

Li,H. (2011) A statistical framework for SNP calling, mutation discovery, association mapping and population genetical parameter estimation from sequencing data. *Bioinformatics*, **27**, 2987-2993.

Li,H. and Durbin,R. (2009) Fast and accurate short read alignment with Burrows-Wheeler transform. *Bioinformatics*, **25**, 1754-1760.

Tan,A., *et al*. (2015) Unified representation of genetic variants. *Bioinformatics*, **31**, 2202-2204.

Tarasov,A. *et al*. (2015) Sambamba: fast processing of NGS alignment formats. *Bioinformatics*, **31**, 2032-2034.
