# Supplementary material for: SVScore: an impact prediction tool for structural variation
Source: Bioinformatics. 2017 Jan 3;33(7):1083–5. doi: 10.1093/bioinformatics/btw789 (PMC5408916; doi:10.1093/bioinformatics/btw789)
Supplement: Supplementary Data [file btw789_supp.zip › SVScore_Bioinformatics_SupplementaryData.docx]

**
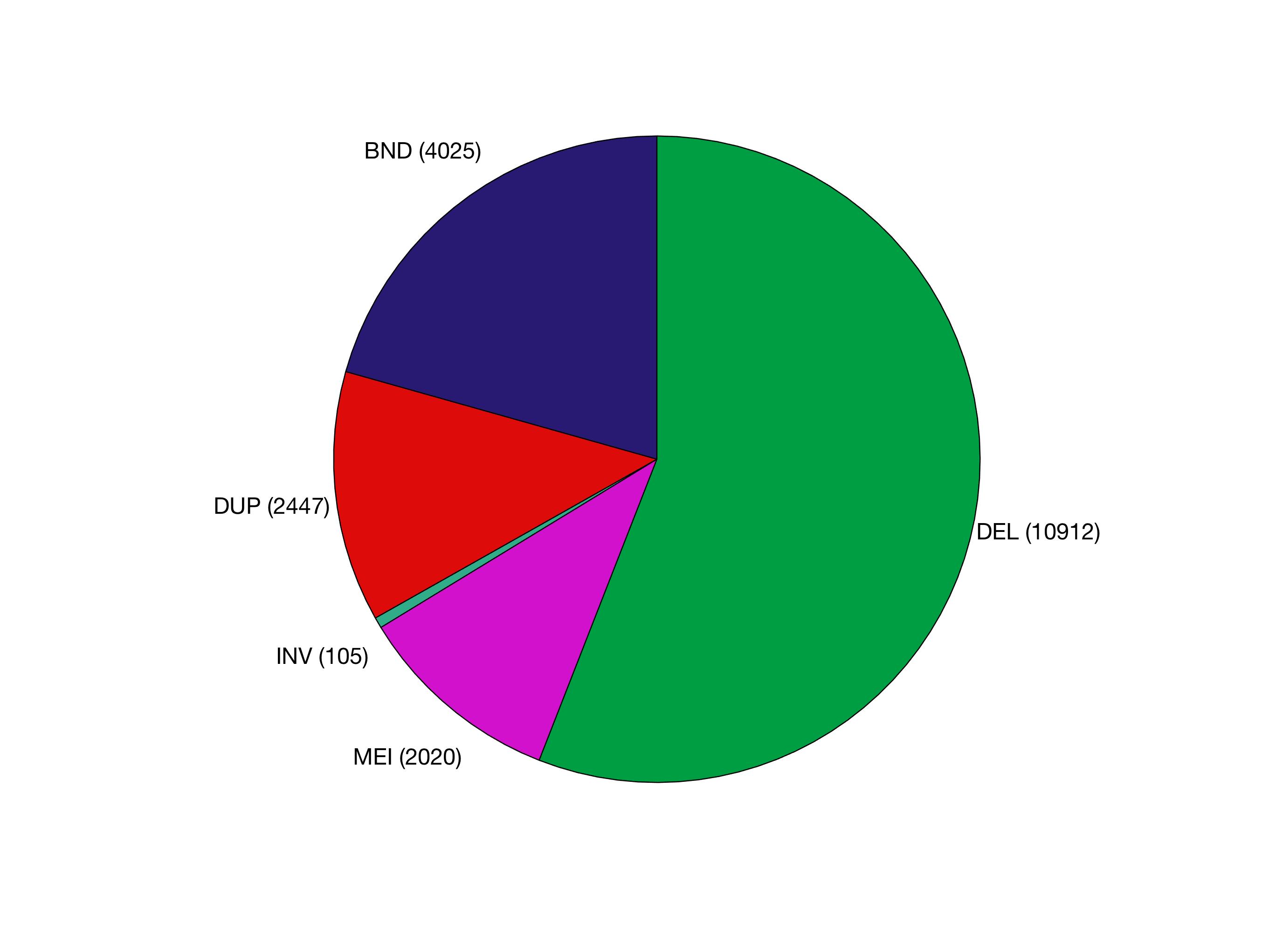
**

**Supplementary Fig. 1. Variant type composition of SV callset**. INV – inversion, MEI – mobile element insertion, DEL – deletion, INS – novel sequence insertion, DUP – tandem duplication, BND – unclassified novel adjacency.

**Supplementary Fig. 2. Allele frequency skew of high-scoring structural variants.** Variants are separated first into classes based on impact score percentile, then into allele frequency bins. Each impact score class is normalized to 1 so that the height of each bar represents the fraction of the given impact score class that is in the given allele frequency bin.


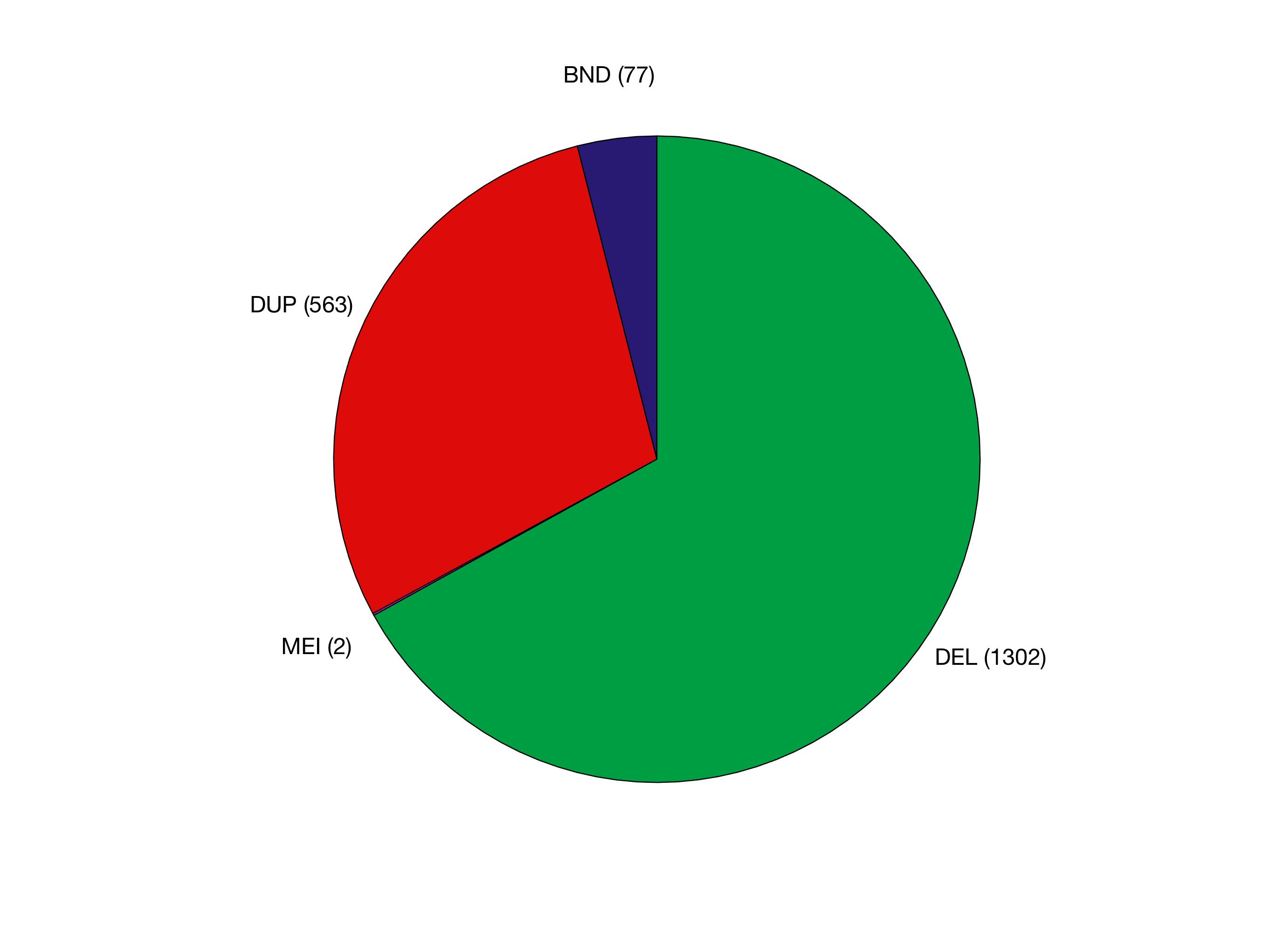


**Supplementary Fig. 3. Variant type composition of SVs at or above the 90^th^ percentile of impact scores**. DEL – deletion, MEI – mobile element insertion, DUP – tandem duplication, BND – unclassified novel adjacency

**Supplementary Fig. 4. SV distributions separated by SVScore impact scores.** SVs in each plot were placed into logarithmically sized length bins and plotted as histograms. **(a)** All SVs. **(b)** Benign SVs – impact scores below the 50^th^ percentile. **(c)** Intermediate SVs – impact scores below the 90^th^ percentile and at or above the 50^th^ percentile. **(d)** Pathogenic SVs – impact scores at or above the 90^th^ percentile.

**Supplementary Fig. 5** Variant score distributions. **(a)** Distribution of precomputed CADD SNP scores on chromosome 1. The inset shows a zoomed view of the score distribution between 25 and 60. **(b)** SVScore impact score distribution for SVs in phase 3 of the 1000 Genomes Project (blue) and known pathogenic SVs from ClinGen (red). These variants are sampled such that they come from the same size distribution (see Supplementary Methods). Irregularities around 30 in the impact score distribution are likely a result of the irregularity in the CADD distribution at the same score (shown in the inset of panel **(a)**). **(c)** Same as **(b)**, but comparison is made with FinMetSeq, the same 950 Finnish genome data set used for analysis in the main text of the paper. The red distributions differ from one another in that panel **(b)** is sampled so the size distribution matches that of the 1000 Genomes Phase 3 SVs, while that in panel **(c)** is sampled to match the size distribution of the FinMetSeq data set. All variants above 1 Mbp in length were excluded, as SVScore automatically assigns these variants a score of 100.**Supplementary Table 1** – Performance statistics, in seconds.

|  | Total | Score Extraction | Score Processing |
| --- | --- | --- | --- |
| Preprocessing | 156.7 |  |  |
| LEFT/RIGHT | 0.199 | 0.160 | 0.026 |
| SPAN | 0.485 | 0.134 | 0.320 |
| LTRUNC/RTRUNC | 2.1 per transcript  4.597 per variant | 0.201 | 1.762 |
| Postprocessing | 139.7 |  |  |

**Supplementary Table 2** – Contingency table for SVs at varying impact score thresholds

| SV Percentile | Rare | Common |
| --- | --- | --- |
| Bottom 50% | 2846 | 5420 |
| Top 1% | 180 | 12 |
| Top 5% | 772 | 119 |
| Top 10% | 1528 | 222 |
| Top 15% | 2212 | 351 |
| Top 20% | 2845 | 547 |
| Top 25% | 3449 | 754 |
| Top 30% | 3983 | 1032 |
| Top 35% | 4536 | 1294 |
| Top 40% | 5032 | 1623 |
| Top 45% | 5561 | 1934 |
